# The Extreme Climate Event Database (EXCEED): Development of a picture database composed of drought and flood stimuli

**DOI:** 10.1371/journal.pone.0204093

**Published:** 2018-09-20

**Authors:** Sabrina de Sousa Magalhães, Diana Kraiser Miranda, Débora Marques de Miranda, Leandro Fernandes Malloy-Diniz, Marco Aurélio Romano-Silva

**Affiliations:** 1 Laboratory of Molecular Medicine, Federal University of Minas Gerais (UFMG), Belo Horizonte, Brazil; 2 Department of Pediatrics, Federal University of Minas Gerais (UFMG), Belo Horizonte, Brazil; 3 Department of Mental Health, Federal University of Minas Gerais (UFMG), Belo Horizonte, Brazil; Universidad de Tarapaca, CHILE

## Abstract

The present study introduces the Extreme Climate Event Database (EXCEED), a picture database intended to induce emotionally salient stimuli reactions in the context of natural hazards associated with global climate change and related extreme events. The creation of the database was motivated by the need to better understand the impact that the increase in natural disasters worldwide has on human emotional reactions. This new database consists of 150 pictures divided into three categories: two negative categories that depict images of floods and droughts, and a neutral category composed of inanimate objects. Affective ratings were obtained using online survey software from 50 healthy Brazilian volunteers who rated the pictures according to valence and arousal, which are two fundamental dimensions used to describe emotional experiences. Valence refers to the appraisal of pleasantness conveyed by a stimulus, and arousal involves internal emotional activation induced by a stimulus. Data from picture rating, sex difference in affective ratings and psychometric properties of the database are presented here. Together, the data validate the use of EXCEED in research related to natural hazards and human reactions.

## Introduction

Worldwide, people are facing disasters caused by climate change. With the increasing number of events caused by global warming (e.g., heavy precipitation events, floods, hurricanes, wildfires and heat waves), more and more people around the world will be affected. Such circumstances affect humans in different ways that could potentially lead to both mental health issues and/or have an impact on a broad range of adversities to the economic, social and environmental field [1–3]. The impact of such climate-related extreme events uncovers vulnerability and exposure of ecosystems and human dynamics [4].

The doubts raised about how to cope with immediate or long-term consequences of natural hazards motivated the development of the Extreme Climate Event Database (EXCEED). The database is proposed as a validated tool for use in a broad range of experimental designs assessing emotional response, with representative stimuli of flood and drought events. Thus, this database could could contribute to insight about how individuals respond to those stimuli.

The use of well-designed instruments that are reliable and accurate in emotion induction is of paramount importance; still, the selection of stimuli depends on the study design and the targeted empirical answer. EXCEED pictures could be used as emotional triggers to collect and evaluate an extensive set of responses for both healthy and clinical samples. For instance, experimental approaches in psychophysiological research with EXCEED stimuli could be directed to measuring facial expression, physiological reactions (e.g., peripheral physiological signals or electroencephalographic), behavior accuracy, or cognitive responses (e.g., attention network or memory). Furthermore, the pictures can also stimulate investigations about beliefs and actions related to climate change and the role of media and arts in mediating behaviors and perceptions. Another experimental use for the database is for those who need psychotherapeutic intervention after a natural disaster experience, including the traumatic ones. In the latter case, the literature consistently suggests that there are immediate and long-term consequences for mental health and human development after natural disasters [5–7]. Therefore, we expect that these inquiries assist in the creation of important public health policies and mitigation actions, especially in socially disadvantaged populations [7,8].

The development of sets of standardized stimuli requires a representative theory of emotion and affect. First, emotional responses arise after the evaluation of meanings, pertinence, and significance of the stimulus [9]. Simultaneously, the proprioceptive feedback modulates the physiological reactions associated with the aroused feeling [10]. Two commonly used perspectives to describe and conceptualize emotions are called dimensional theory and discrete emotion theory [11,12]. The discrete emotion theory claims that there is a set of basic emotions that triggers specific behavior and physiological responses. Proponents of this theory have concluded that there are six basic discrete emotional states that emerge from a cognitive appraisal of the environment: fear, disgust, surprise, anger, happiness and sadness [13].

Still, the model of emotion as a cognitive operation matched to internal physiological changes is the basis for the proposition of a dimensional perspective of emotional response. The dimensional approach to emotion postulates that the emotional response arises from basic motivational processes, mainly organized under the influence of valence and arousal [11].

However, initially, three-dimensional aspects were responsible for the organization of the core affect: a) valence, a contrast between states of pleasure and displeasure; b) arousal, that compares states of agitation and relaxation; and c) approach-avoidance state, also known as “motivation direction”, which compares the tendency to approach versus the tendency to avoid a stimulus [12]. Further development of the dimensional perspective leads to the circumplex model of affect. This model postulates that all affective states are linear combinations of the two primary neurophysiological systems: valence (a pleasure-displeasure continuum, also referred to here as positive-negative continuum) and arousal (an excited-relaxed continuum, also known as activation or alertness), along with cognitive consideration and labeling of the experience [14–16]. In this model, valence is one of the fundamental properties of the core affect [17, 18], comprising the cognitive process of evaluating the environment and coding it according to pleasantness or unpleasantness. While arousal is the other essential property of all affective stimuli, it encompasses a continuum from relaxed, calm, sluggish, and sleepy to stimulated, distressed, and jittered. The dimensional structure of affect can be plotted in a schematic coordinate axis, where arousal and valence yield two polarities (high and low arousal, and pleasure and displeasure valence, or positive and negative valence). Each emotion can be located in this space, as a linear combination of valence and arousal. Thus, all emotions are formed by the two properties of valence and arousal, and emotional responses can be properly evaluated only through both states. Presently, the level of dominance perspective has been eclipsed by the perspective of the circumplex model of affect.

Despite the theoretical divergence between dimensional theory and discrete emotion perspective, scholars who endorse either paradigm do not consider the theories as incompatible, because human emotion seems to consist of both conscious and unconscious processes [11]. Ultimately, the motivational subsystems from valence/arousal underlie discrete emotional states. However, to our purpose, the dimensional perspective provides meaningful ways to access emotion without the need for one's inherent ability to conceptualize and verbalize distinct emotional states. In addition, evidence indicates that the way an individual labels discrete emotional states varies according to cultural background. Therefore, using valence and arousal ratings enables individuals to avoid the often challenging task of discerning and applying distinct emotional labels to differentiate the emotional experience. Due to individual differences in emotional granularity, it is simpler to report a pleasant or unpleasant feeling and an aroused or calm state instead of nominating emotions [17]. Thus, the dimensional perspective can be a “superordinate theoretical framework” [11] for studying emotional responses.

Previous studies have indicated sex differences in the processing of emotional stimuli and emotion regulation, defined as a series of automatic and controlled responses aimed to modulate the experience and expression of emotion [19]. Know as the female negativity bias hypothesis, women tend to present a stronger reactivity to negative material than men [20, 21]. Thus, the tendency is for the female individuals to rate negative stimuli for a high valence-high arousal responses [22]. On one hand, this trend may explain the vulnerability of females in the increasing rates of anxiety disorders among them [20]. However, a major sensitivity for negative material can contribute to empathy behaviors in the context of suffering and disasters [23].

Currently, there are at least three pictorial databases used as methodological reference for evaluating and classifying emotional responses: the International Affective Picture System, IAPS [24], The Geneva Affective Picture Database, GAPED [25] and The Nencki Affective Picture System, NAPS [26]. All of these databases were validated by using similar dimensional perspective and affective ratings in the domain of valence and arousal on healthy volunteers.

One important remark considering those databases is that all of them built their negative categories mainly upon phobic reactions, scenes of violence and injured or dead human bodies. The lack of a full set of specific natural hazard stimulus is a gap that EXCEED aims to fill. The choice for flood and drought stimuli was influenced by the significant prevalence of these two events worldwide. From 1995 to 2015, floods alone accounted for 47% of all weather-related disasters and, together with droughts events, affected 3.4 billion people (82% of total affected, excluding deaths) around the world [27].

The media coverage of natural hazards is increasing, and the role media plays in society has recently drawn considerable attention. At the time of this writing, the media is a central part of everyday life. Most of the world watched and followed the repercussions of the 9/11 attack on the World Trade Center, the 2004 tsunami in Thailand and the 2005 Hurricane Katrina in New Orleans. The risk associated with the experience of a disaster can be manipulated, amplified, magnified, or minimized by the media [28]. Because disasters are commonly discrete events, the media often covers severe disasters such as floods, hurricanes and earthquakes. Less attention is given to chronic conditions, especially droughts, because of their slow onset and broad spatial extent, unless they become extremely critical [29]. A worldwide effort to consider drought as a significant disaster and to protect the drought-vulnerable populations has already begun, for example, by agencies within the World Health Organization and United Nations [27].

The objective of this study was to present the development and validation of a broad set of normative, color photographs, emotional stimuli for experimental investigations of emotion in the context of climate associated disasters. We additionally aimed at investigating sex-related differences in affective ratings, expecting a high valence–high arousal sex effect in participants of female gender [19–23].

## Materials and methods

All procedures of this study were approved by the appropriate local ethics committee (CAAE: 26886814.9.0000.5149). For a summary of the Method, see S1 Fig in the Supporting Information, which describes the main methodological steps used.

### Material

EXCEED is freely accessible to the scientific community for noncommercial use, and it is available at: <https://doi.org/10.6084/m9.figshare.4602016.v1> [30]. Each image within the database receives an abbreviation identification letter for the category that it belongs to, followed by a crescent number within the group, the respective attribution credit and creative common (CC) license. The letter N represents neutral pictures (range: N01 to N50), FL designates the flood group (range FL01 to FL50), and DR designates drought pictures (range DR01 to DR50). All pictures are in JPEG format; however, the resolution and dimensions could vary between pictures. Because the aim was to pursue an ecological validity of the pictures, the physical properties of the images were not controlled. This is examined in the Discussion section as it may restrain the applicability in some methodological platforms. All EXCEED images are under CC license, and each image is under specific license according to restrictions modifications, and use in commercial settings (information regarding credits of the images and respective licenses can be consulted at S1 Table).

Pictures were selected mainly through an extensive online search of CC license repositories (e.g., Wikipedia Commons, Flickr—constraining the search for online CCs images, Empresa Brasil de Comunicação—a public Brazilian media agency that use CCs license for all the images provided). Social media sites like Facebook were searched too. We only used images from Facebook if we could contact the owner, explain our aims and receive written consent to use the images. The vast majority of the photos were retrieved from Flickr. Some images were free to modify, so ten pictures receive subtle alterations. CC licenses require that the user must indicate if alterations were made, thus in compliance with CC license requirements, we list in a supplementary file all information demanded to be in accordance with international guidelines (S1 Table).

EXCEED database included two negative categories (flood and drought) of images that, virtually, could culturally resemble any worldwide reality related to those events. Inclusion criteria for these weather-related categories were that a picture must either portray a realistic event related to the topic, such as a scenery affected by an extreme event (urban or rural), or depict living beings in that context. Pictures were from all over the world, including Africa, Brazil, India, and the United States, among others. For the neutral category, inanimate objects were chosen, since finding scenes of people with neutral facial expressions was challenging [25]. Initially, 50 pictures were selected for each category. Consequently, the dataset at first consisted of 150 pictures in both in landscape or portrait orientation. Our hypothesis postulated that drought and flood pictures should exhibit valence of 1 or 2 (interpreted as negative valence) and arousal of 4 or 5 (indication of high arousal) and that the control pictures should be rated with 3 for both valence and arousal (neutral valence and neutral arousal). High valence and low arousal pictures were supposed to represent positive and pleasant images, which did not apply to this database. Then, we ran preliminary analysis to identify any image that received inadequate ratings because of their unlikely or unpromising representation of a particular category so that none of the pictures could be considered an outlier. The criterion adopted to search for outlier pictures can be found in the Results section, although none picture were identified as an outlier.

### Participants

For the validation process, a convenience sample of 50 healthy Brazilian volunteers participated in the study (25 women). They were recruited through online and social media advertisements. We sought volunteers by communicating with our acquaintances, family and friends.

A total number of 70 volunteers began to rate the pictures, but only 50 completed the survey. Because approximately 30% of individuals dropped out, and in order to not overweight the initial ratings versus the final ones, we decided to remove all individuals who did not reach the end of the experiment. Thus, we do not have missing data, because responses to all the questions were required for the development of the experiment.

The final sample of 50 individuals had a mean age of 36.94 years (ranging from 20 to 69 years; *SD* = 13.12). Minors were not included in the study. Most participants had at least one academic degree (78%) and had not personally experienced flood or drought conditions (62%), although fourteen participants (28%) reported facing flood incidents, one reported experiencing a drought event (2.0%), and four (8.0%) reported experiencing both events. Due to the small number of individuals who reported previous personal experience with natural hazards and the impracticable possibility of inferential analysis within such subgroups, the final sample consisted of all participants regardless of their experience.

### Statistical analysis

The Statistical Package for the Social Sciences platform was used to perform statistical analysis. Descriptive analysis indicated demographic profile of the sample, valence and arousal means and standard deviation rates. We use inferential analysis to verify differences between the EXCEED categories through ANOVA and *post hoc* tests, as well as examine sex-related differences in affective ratings. If significance differences were obtained, we estimated the effect size value through Eta Squared indices due to the possibility of bias because of the small sample. In addition, one limitation of ANOVA is the lack of magnitude difference indicated by the test. Effect size indicates how much variance in the dependent variable is due to the variance of the independent variable.

The association between valence and arousal is an important discussion in the field. To verify the strength and direction of the association, Pearson correlation coefficient (*r*) was calculated. Generally, *r* values between 0.1 and 0.3 indicate a small correlation, values between 0.3 and 0.5 indicate a medium to moderate correlation, and *r* values more than 0.5 indicate a strong correlation [31], although some authors argue that only *r* values above 0.7 should be considered strong correlations [32].

The quality of the EXCEED data relies on empirical evidence for psychometric properties. We elected two distinct methods to run the analysis. Cronbach’s alpha provides information about the homogeneity of the database. On the other hand, the split-half method enabled conclusion about the internal consistency and we split the database, comparing the first half of items versus the second half of items, because we want to ensure that habituation or sensitization did not occur while experiment was in progress.

### Procedures

The validation processes comprised rating each picture for valence and arousal, because we considered them as fundamental properties of affect. Thus, the level of analysis was the subjective experience accessed through self-report. The chosen method (described below) was similar to other picture databases, such as IAPS [24], GAPED [25], and NAPS [26].

The Survey Monkey online software was used to collect data through a link made available via email, acquaintances, and social media. Before stimuli presentation, volunteers read a briefing of the research and the free and clarified consent terms, then indicated with selection of a box the agreement to participate. After this introduction and only with the authorization box chosen, the next page presented comprehensive instructions, with examples, regarding the task and rating scales. They were introduced to the procedures of rating and became familiarized with the dimensions and instruments before the experiment.

**Fig 1:**
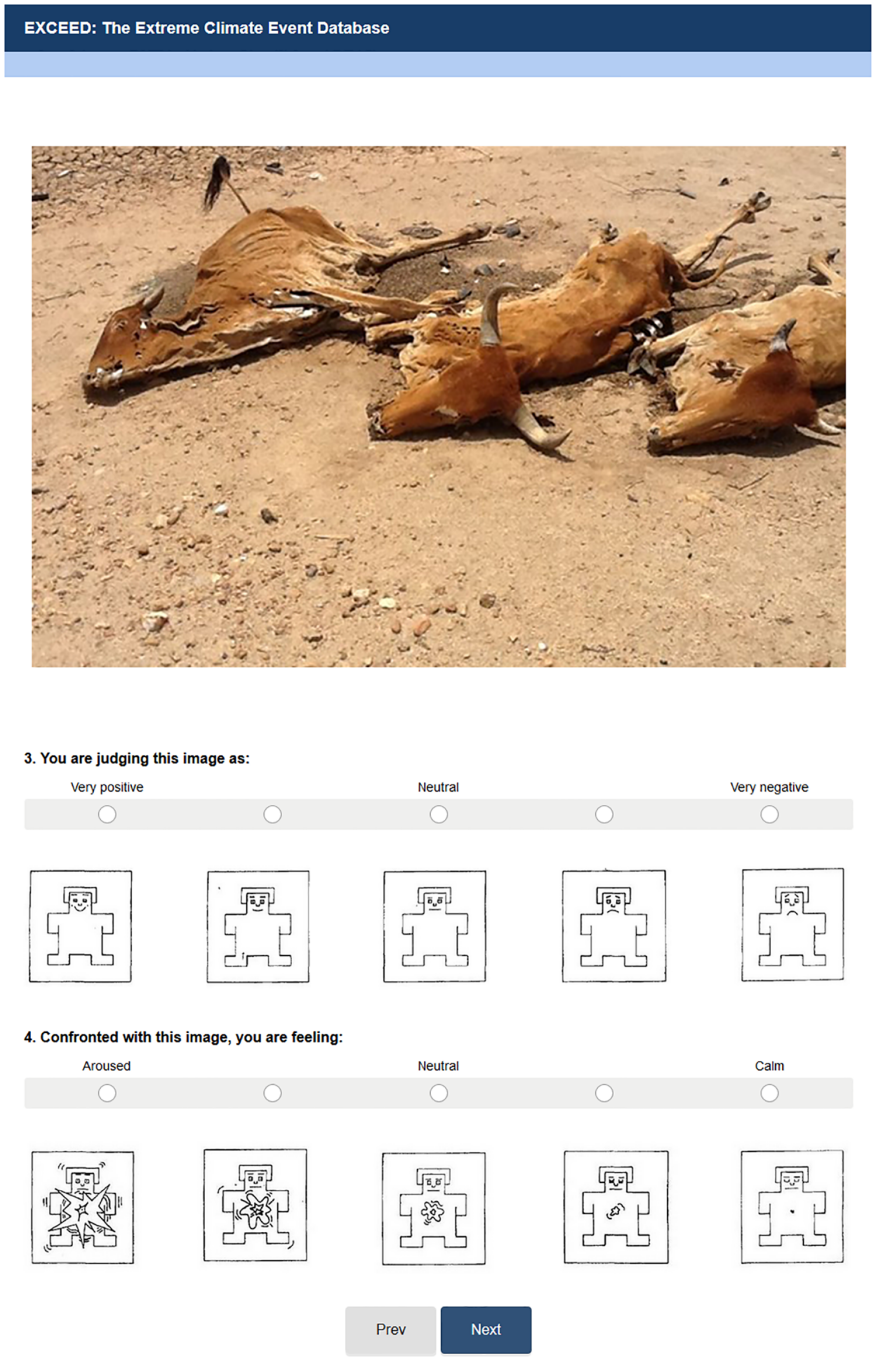
An example of image presentation and respective ratings of valence and arousal for EXCEED validation. The language portrayed is English, but the original language was Brazilian Portuguese. The Self-Assessment Manikin (SAM) system was presented together with two five-point scales. The valence scale (the first question) went from very positive to very negative. The midpoint represented a neutral condition. For rating, participants were instructed to complete the sentence "You are judging this image as:”. The arousal scale (the subsequent question) was presented with the sentence “Confronted with this image, you are feeling:” and ranged from aroused to calm. The midpoint matched a neutral state. Instructions paralleled those described by Dan-Glauser and Scherer [25]. (Image portrayed is DR32 by Padre Djacy Brasileiro / CC BY-NC-ND 2.0, from EXCEED).

EXCEED pictures were presented in a semi-randomized order, with the constraint that no image belonging to the same category occurred consecutively [25]. Among the volunteers, the presentation order was constant for all participants, as a limitation of the chosen software. For each picture presented, the participant rated them for valence and arousal, and after completing the requisite ratings for one image, a button at the end of the screen allowed the participants to move to the following page with the subsequent stimulus (Fig 1).

To obtain an affective rating of the pictures, we used two strategies: a five-point graded verbal scale (one to valence ratings and another to arousal ratings) and a pictorial scale based on the SAM. The valence five-point scale went from very positive to very negative, with the midpoint representing a neutral condition; participants provided ratings by completing the sentence: "You are judging this image as…". The arousal scale was presented with the sentence "Confronted with this image, you are feeling…" and ranged from aroused to calm, with the midpoint indicating a neutral state.

SAM is a picture-oriented instrument that includes five cartoon-like figures for the valence and arousal dimensions. SAM is a widely used system to measure emotional responses to a variety of stimuli, including images and sounds [33]. For the valence dimension, SAM ranges from a smiling and happy face to a desolate and unhappy one. When representing the arousal dimension, SAM varies from an excited, wide-eyed figure to a sleepy figure with eyes closed. The participants were instructed to choose from any of these five images associated with the labels from the verbal scale above each SAM's figure. For valence ratings, we equated the internal continuum of pleasure-displeasure with the positivity or negativity of the picture presented and rated by individuals. SAM was selected due to its validity in determining the subjective experience of emotion related to the appraisal of the stimuli. Its applicability covers a variety of populations, including children and people with less education [34]. Studies have shown that ratings of valence and arousal using this method are highly correlated with affective measures obtained by semantic scales of emotional stimuli [35].

Fig 1 depicts a print-screen of a page of the experiment, in which the EXCEED picture was presented to the upper margin and centralized, followed by the question about valence, the five-point scale lined to SAM scales, and arousal investigation. Although the original language of the experiment was Brazilian Portuguese, Fig 1 displayed a translated version of the survey questions. The instructions followed those described by Dan-Glauser and Scherer [25].

The experiment lasted about forty minutes, but time varied according to each individual´s rating speed, since there was no time limit. After data collection, the answers for valence and arousal ratings were replaced with numbers (range 1 to 5). The very positive and aroused choices were coded as 5, the subsequent choices as 4, the neutral conditions as 3, the following choices as 2, and very negative and calm as 1. Thus, higher numbers indicated positive valence or greater arousal.

## Results and technical validation

For each of the 150 pictures, mean value ratings were obtained for both valence and arousal categories. The ratings ranged 1 to 5 and higher numbers indicated positive valence and greater arousal. A score of 3, which indicates the middle point of the scales, showed a neutral condition which could apply for both affective dimensions. First, we conducted exploratory analyses to determine whether any picture was rated differently than was expected for their respective group—for instance, if a drought picture received neutral valence and/or low arousal, whereas the hypothesis supported a negative valence and high arousal. Nearly of all the drought and flood pictures should exhibit valences of 1 or 2 and arousals of 4 or 5, and the control pictures should be rated a 3 for both valence and arousal. Therefore, to obtain unambiguous categories and images that depicted the emotion intended, outliers were investigated. The criteria adopted were the mean plus 1.5 standard deviation for valence ratings, the mean minus 1.5 standard deviation for arousal ratings in flood and drought categories, and the mean plus and minus 1.5 standard deviations for both valence and arousal in the neutral category. Nevertheless, there was no outlier picture in our database. Therefore, the final database contained the same 150 images.

**Fig 2:**
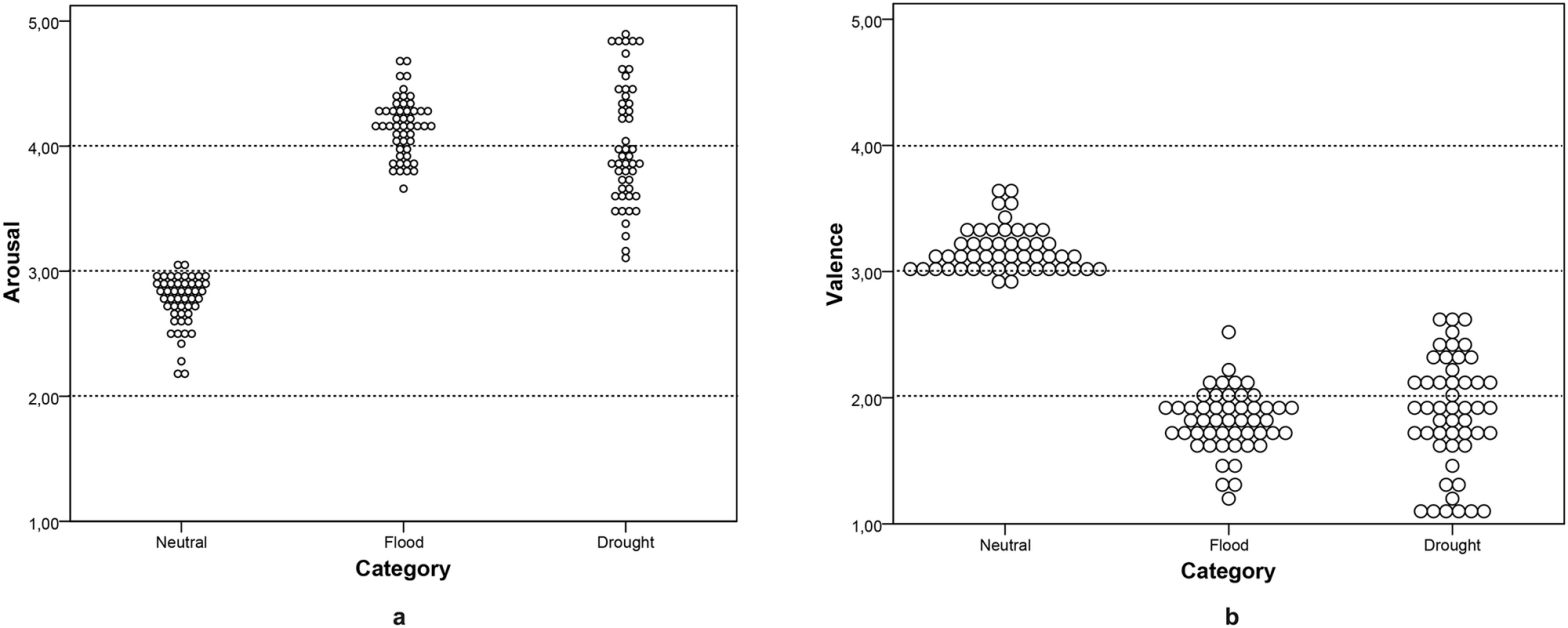
Arousal (a) and valence (b) distribution of outcome ratings for each category. In the neutral category, the distributions of rating indicated a neutral condition for both affective dimensions. The weather categories were rated with higher arousal and with negative valence, yet the Drought distributions were more spread-out than Flood's. Each dot in the graphic represents a picture.

Descriptive statistics for both valence and arousal dimensions for all EXCEED pictures were presented as Supporting Information (S1 Table). Table 1 summarizes the main features of each category group and shows the values for women and men separately. Group difference was observed between the natural hazards conditions compared to the neutral, for both dimensions (valence: *F* (2,147) = 313.58, *p*< .001; arousal: *F* (2,147) = 264.56, *p*< .001), but not between drought and flood pictures. A Bonferroni *post hoc* test indicated that flood and drought pictures were more negative than neutral and elicited more arousal in individual participants. Fig 2 depicts the distribution of the outcome ratings in valence and arousal for each EXCEED category.

**Table 1:** Descriptive statistics for valence and arousal, organized by category groups and by sex.

Category	Valence	Arousal	Women		Men	
			Valence	Arousal	Valence	Arousal
	*M* (*SD*)	*M* (*SD*)	*M* (*SD*)	*M* (*SD*)	*M* (*SD*)	*M* (*SD*)
**Neutral**	3.17 (0.50)	2.76 (0.66)	3.18 (0.53)	2.73 (0.70)	3.16 (0.48)	2.79 (0.61)
**Flood**	1.81 (0.70)	4.16 (0.76)	1.66 (0.65)	4.36 (0.72)	1.96 (0.72)	3.96 (0.75)
**Drought**	1.87 (0.78)	4.03 (0.91)	1.76 (0.79)	4.21 (0.91)	1.97 (0.76)	3.85 (0.87)

Emotion research usually describes sex differences in emotional processing toward a stronger physiological and cognitive response displayed by women compared to men [21, 36]. For the EXCEED, women and men responded slightly differently in affective ratings for the flood and drought category stimuli (*p*< 0.001). Women exhibited a tendency to rate the pictures in a more negative way and reported more feelings of disturbance when confronted with the stimuli, although the effect sizes were small (ɳ^2^ = 0.22 for valence and ɳ^2^ = 0.27 for arousal in flood category; ɳ^2^ = 0.15 for valence and ɳ^2^ = 0.20 for arousal in drought category).

**Fig 3:**
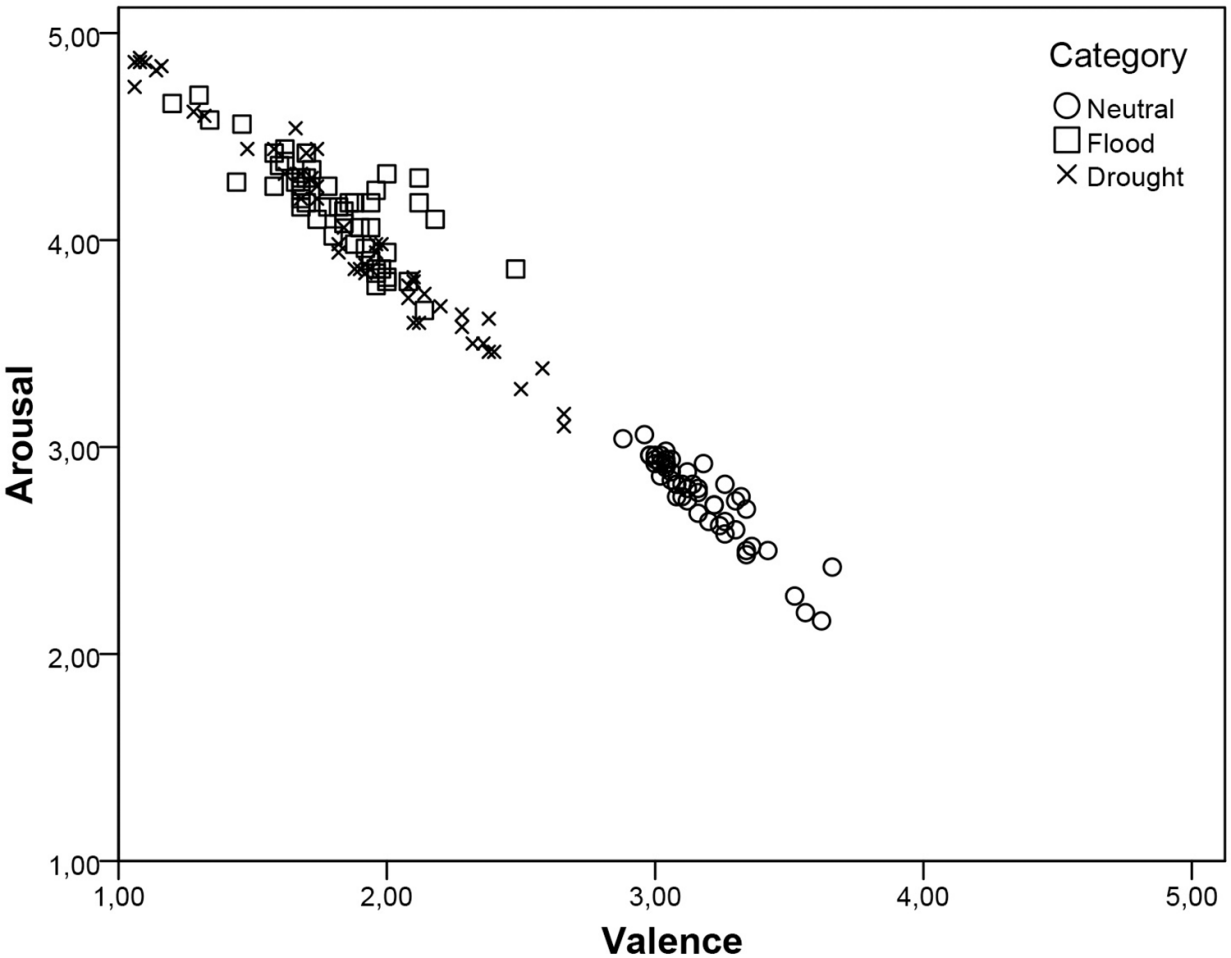
The relationship between arousal and valence rating of EXCEED pictures in affective space. Each dot in the graphic represents a picture. The dimensions exhibited a robust negative linear relationship.

Considering all pictures in the database, strong correlations were obtained for arousal and valence (*r* = -0.82, *p* < .001), which was expected in order to enhance the validity of the database. For each category, the same moderate to strong correlation pattern was observed between affective dimensions (neutral: *r* = -0.69, *p*< .001; flood: *r* = -0.59, *p*< .001; drought: *r* = -0.76, *p*< .001). Fig 3 shows the distribution of valence and arousal for EXCEED pictures in the affective space.

Finally, an internal consistency analysis was conducted with two distinct methods and the results are presented in Table 2. The two methods adopted indicated robust and convergent indices. Therefore, empirical evidence for the internal validity and reliability of the database was gathered.

**Table 2:** Internal consistency analysis for EXCEED, according to categories, affective dimensions and method used.

Category	Affective dimension	Method	
		Cronbach’s alpha	Split-Half
**Neutral**	Valence	0.89	0.83
	Arousal	0.94	0.91
	Both dimensions	0.76	0.80
**Flood**	Valence	0.96	0.93
	Arousal	0.98	0.95
	Both dimensions	0.78	0.91
**Drought**	Valence	0.94	0.89
	Arousal	0.95	0.89
	Both dimensions	0.65	0.77
**EXCEED**	Valence	0.96	0.91
	Arousal	0.96	0.91
	Both dimensions	0.80	0.87

## Discussion

This study aimed to develop and validate a picture database that may ecologically represent the environmental realism associated with drought and flood. Emotion arises from a complex interaction between cognitive appraisal and neurophysiological changes, and it is related to valence and arousal systems, including distinct neural systems underlying each affective dimension [14, 37]. The valuation of surrounded stimuli is made continuously and automatically, enunciating something about its relevance and value. This internal valuation processing produces changes in core affective states, i.e., the emotional neurophysiological state experienced as valence (pleasant or unpleasant) and as arousal (activated or deactivated). Core affect gives information about the relationship between the individual and its current environment at a given point in time. Therefore, core affect provides a standard metric for comparing different events [38].

For all EXCEED pictures, participants were instructed to rate according to their personal reactions to the image. The affective rating obtained supported the hypothesis raised about the classification of the pictures. Even though the majority of the subjects did not personally experience extreme events, climate change awareness occurs via different media, politics and arts, which presumably primed the individuals for the predicted emotional reactions. After all, the induction of real affect is favored by stimuli that are pertinent to the individual. However, higher sample variability and different affected/non-affected status of participants must be important to further evaluate the potential use of the tool. Furthermore, the potential role of media interference in emotional reactions must be better characterized.

One important outcome was the successful differentiation between weather categories and the neutral group. They were rated more as displeasureable and more disturbing than neutral pictures. Of the 150 initially chosen pictures, all achieved the inclusion criteria. Although the drought category contained pictures with most variability within the group (see Fig 2). This pattern could be explained by the characteristics of the sample used for the affective ratings. The semi-arid vegetation, usually associated with dryness depicted in some pictures in the drought category, is, in fact, a familiar and ordinary landscape commonly seen in the Brazilian northeast coast or even in cities without scarce water supplies. On the other hand, any picture that represented flood conditions seems to be more arousing and with negative valence for Brazilian individuals. Another possible mechanism underlying these data is the common belief that drought is not a disaster itself, an affirmation supported by the frequent absence of media coverage about it [29]. Due to its chronic nature, people tend not to consider drought as a natural hazard compared with acute events such as tornados, tsunamis or earthquakes. Another important observation is that some pictures depicted disaster, but others depicted rescue teams. Therefore, they had an intrinsic positive aspect within the harshness of the scene. The perception of individuals about the considerations previously delineated must be better examined to test this hypothesis. Furthermore, validation with distinct demographic conditions would also help to elucidate the findings.

Sex differences in rating emotional stimuli were observed. Women tended to react more and to rate pictures with more arousal and valence than men did; however, this finding only held for the extreme conditions. There were no differences in neutral ratings. These finding are in accordance with the female negativity bias hypothesis [20] and despite the fact that the two-gender sample size was small, the same pattern was also observed in other studies, including those with physiological measures that indicate a sex-distinct processing of emotional content [19, 21, 22, 26].

The relation between valence and arousal is an important issue in the organization of the human affective system. It has been consistently found that increased arousal is associated with positive and negative valence extreme values [18, 21, 24, 26, 33, 35]. Our findings support this pattern of association between valence and arousal, as the negative valence pictures were associated with increasing arousal ratings.

The negative linear relationship between the two affective dimensions considered that the emotional experience should be regarded as a continuum, ranging from a neutral state to an unpleasant distressed feeling. The data suggest that to analyze affective experience, both valence and arousal should be considered, as they maintain a close linear relationship. The high correlation observed (*r* = -0.82, *p* < .001) was expected and described in other experiments validating pictures in databases studies. Marchewka et al. [26] found a significant negative correlation between valence and arousal, including an index of -0.90. In a Brazilian sample, IAPS was validated by Ribeiro et al. [35] and they also observed a correlation of -0.82 for valence and arousal.

Another empirical evidence to validate EXCEED was obtained through internal reliability data. Both Cronbach’s Alpha and split-half methods demonstrated convergence between indices. Beside the investigation of item’s consistency provide by the former, the latter method enable the investigation about any effect resulted from the progression of the experiment. The analyses did not suggest the occurrence of habituation or sensitizations, due to the consistency of the indices. Hence, it revealed coherent ratings and congruent construct measures across the categories, affective dimensions and with the entire database, providing evidence for both internal validity and reliability.

The present study has some limitations that might affect the generalizability of the described data. Given the complexity of the theme and some methodological limitations, we need to interpret the results carefully. The data from EXCEED was obtained from a small, healthy, highly educated Brazilian adult population and ratings from other distinct populations must be obtained. After all, emotional experience also differs across age, developmental stages, and cultural context. Children, for example, exhibit a limited capacity to describe their affective state, having a tendency to cluster emotions under more generic labels [39]. This lack of differentiation of affective states leads to a circumplex model of affect poorly differentiated in children, mainly grouped to extremes of valence and less discriminative for the arousal dimension.

The majority of the sample is from Minas Gerais state (78%), which is a Brazilian state where both natural hazards considered in the study frequently occur. So, participants have familiarity with drought and flood. Although we tried to expand our sample to other Brazilian states and even increase the number of individuals, the procedure of rating the images was considered unfriendly by the participants and we did not succeed in doing so.

One can argue that sensitization occurred along the presentation of pictures for affective evaluation. This phenomenon arises when the continuous presentation of unpleasant images leads to an increasing aversive impact on the individuals [40] and, most commonly, it is not observed for neutral or pleasant pictures. As a result of the lack of randomness in the presentation of the images to participants, it may lead to more susceptibility to fatigue or over/underestimation of the stimulus. On the other hand, some studies have found that affective discrimination is maintained across trials [40], suggesting that emotional reactions and emotional appraisals do not habituate or become desensitized across multiple presentations of similar emotional material. In fact, Dan-Glauser and Scherer [25] verified that, in general, their ratings were not influenced by the duration or sequential presentation of the stimulus. Thus, the impact of sensitization across images could be attenuated considering those pieces of evidence. In addition, data regarding reliability implied an important stability of the database across picture presentation, which contrasts with the claim that sensitization or habituation may occurred in our experiment.

As our database represents mainly disaster pictures, it was expected to impact the participants, although a proper physiological investigation of this question has to be undertaken. After all, the notion that arousal dimension directly represents sympathetic activation has been challenged [9]. One of the arguments is that the affective rating might be dissociated from the actual corresponding feeling state, although this issue could be more prominent for arousal than for valence. On the other hand, others claimed that even briefly presenting affective pictures can activate emotional responses [39]. An association between the two methods, i.e., self-report measures and physiological markers of valence and arousal, would be ideal.

Additionally, EXCEED neutral category was composed of inanimate objects, because we were not able to find enough pictures with neutral content. In a pilot study (data not shown), we found that even a neutral landscape will be considered positive to participants, likely due to a spontaneous comparison between scenes portrayed in flood/drought conditions and supposed neutral ones. Furthermore, images picturing people with neutral facial expression are challenging to find. The deliberate decision to use inanimate objects for the neutral category could, unfortunately, bias the emotional appraisal favoring the negative ones. However, we do not expect that this claim could affect our data in a suggestive manner. First, the distributions of ratings for the database (Fig 2) indicated an important internal variability. Second, some pictures in the database represent only landscapes affected by a dry weather or a flood event, and even those were rated with negative valence and high arousal.

Finally, the last limitation was that the EXCEED evocative stimuli pictures are inherently heterogeneous regarding the number of elements depicted, orientation, and illumination compared to image sets deliberately arranged (e.g., posed face photographs). Those features could influence the appraisal of the pictures. However, the aim of the database was more to represent a realistic scene with ecological validity than to have strict parameters of the stimulus. Therefore, no constraint was imposed on physical properties (e.g., luminosity, contrast, quality of the image) and no standardization rules were even set for the images. Those properties could impose significant limitations for use in research with electroencephalography (EEG) techniques, for example, which are important to provide external validity to the database. However, again, the focus was to create a database with ecological validity. Furthermore, the applicability and the purpose of the database are beyond EEG-like methodologies. For example, in a recent meta-analysis and systematic review, Brown et al. [41] found that cognitive behavioral therapy is one effective intervention recommended for traumatized children and adolescents. EXCEED pictures can provide resources aligned to this treatment. In addition, experimental designs aimed to investigate emotion regulation, the impact of mental imagery, psychophysiological response in clinical samples (e.g., PTSD patients) or healthy individuals can take advantage of the validated EXCEED database.

## Conclusion

A standardized rated affective database supports better-controlled experiments of emotional stimuli, as well as allows comparisons across studies, including those with different research settings. Studies need to carefully choose the type of pictures and their value prior to testing and controlling in order to obtain a valuable inducing material [25]. In our study, data for valence and arousal was obtained for each picture and had the subjective experience assessed through self-report. Valence and arousal displayed a negative linear relationship, and all dimensions were highly correlated. Finally, EXCEED exhibited higher values for internal validity and reliability. The psychometric properties of the data implied a consistency, stability and validity of the entire database. Thus, we gathered empirical evidence to validate the EXCEED database for use in psychophysiological and social research related to climate-associated extreme events. Although these are limited findings that we intend to replicate in a multicultural background and a more diverse sample, to our knowledge, this is the first database specifically designed to attend a worldwide climate change demand in the field of affective research and natural hazards.

## Supporting information

S1 Fig Methodological flowchart of EXCEED development and validation. (PDF)

S1 Table Characterization of EXCEED pictures, organized by category. Attribution credit, licenses, original URLs, an indication of modifications, and ratings on valence and arousal for each EXCEED picture.
(DOCX)
