# Supplementary material for: The Extreme Climate Event Database (EXCEED): Development of a picture database composed of drought and flood stimuli
Source: PLoS One. 2018 Sep 20;13(9):e0204093. doi: 10.1371/journal.pone.0204093 (PMC6147476; doi:10.1371/journal.pone.0204093)
Supplement: S1 Fig — (PDF) [file pone.0204093.s001.pdf]

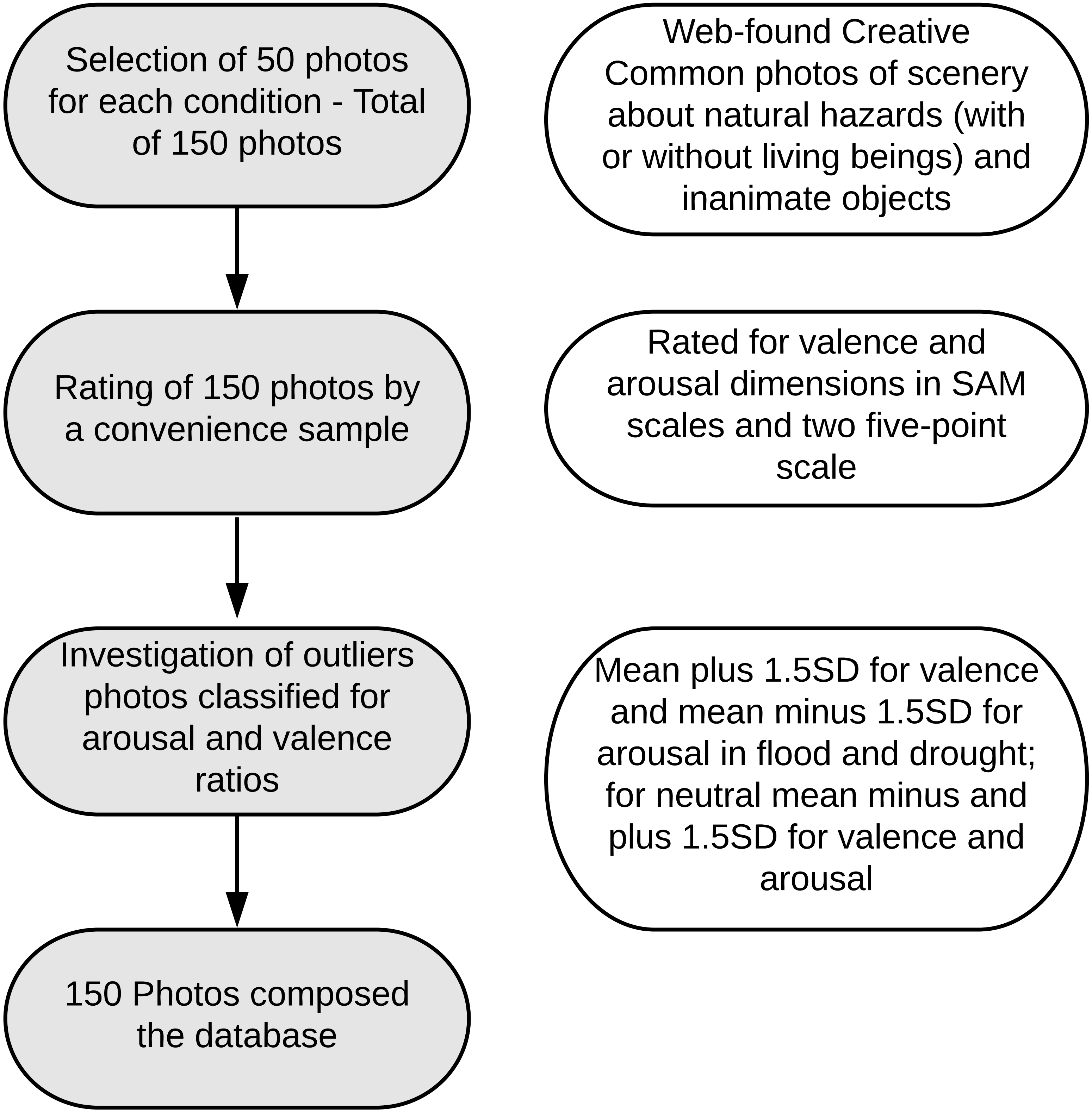
Selection of 50 photos for each condition - Total of 150 photos
Web-found Creative Common photos of scenery about natural hazards (with or without living beings) and inanimate objects
Rating of 150 photos by a convenience sample
Rated for valence and arousal dimensions in SAM scales and two five-point scale
Investigation of outliers photos classified for arousal and valence ratios
Mean plus 1.5SD for valence and mean minus 1.5SD for arousal in flood and drought; for neutral mean minus and plus 1.5SD for valence and arousal
150 Photos composed the database
