# Supplementary material for: The Extreme Climate Event Database (EXCEED): Development of a picture database composed of drought and flood stimuli
Source: PLoS One. 2018 Sep 20;13(9):e0204093. doi: 10.1371/journal.pone.0204093 (PMC6147476; doi:10.1371/journal.pone.0204093)
Supplement: S1 Table — Attribution credit, licenses, original URLs, an indication of modifications, and ratings on valence and arousal for each EXCEED picture. (DOCX) [file pone.0204093.s002.docx]

**S1 Table. Characterization of EXCEED pictures, organized by category.** Attribution credit, licenses, original URLs, an indication of modifications, and ratings on valence and arousal for each EXCEED picture.

| **Picture identification** | **Category** | **Descriptive statistics** | | **Credits** | **License** | **URL** | **Modifications** |
| --- | --- | --- | --- | --- | --- | --- | --- |
|  |  | **Valence** | **Arousal** |  |  |  |  |
|  |  | ***M* (*SD*)** | ***M* (*SD*)** |  |  |  |  |
| N01 | Neutral | 3.32 (0.55) | 2.76 (0.72) | Philippa Willitts | CC BY-NC 2.0^a^ | https://goo.gl/ja6rgg | - |
| N02 | Neutral | 3.34 (0.72) | 2.70 (0.68) | Rafael Acorsi | CC BY-SA 2.0^b^ | https://goo.gl/d2HXH3 | Yes |
| N03 | Neutral | 3.56 (0.61) | 2.20 (0.88) | Ana Guzzo | CC BY-NC 2.0^a^ | https://goo.gl/gjmgXa | - |
| N04 | Neutral | 3.26 (0.60) | 2.64 (0.80) | Raúl Hernández González | CC BY 2.0^c^ | https://goo.gl/wL9Po1 | Yes |
| N05 | Neutral | 3.52 (0.81) | 2.28 (0.95) | Rene de paula jr | CC BY-NC-ND 2.0^d^ | https://goo.gl/GCFQT2 | - |
| N06 | Neutral | 3.06 (0.42) | 2.94 (0.31) | Richard Garside | CC BY 2.0^c^ | https://goo.gl/waCU8b | - |
| N07 | Neutral | 2.88 (0.52) | 3.04 (0.64) | Samuel M. Livingston | CC BY 2.0^c^ | https://goo.gl/vkMmF4 | - |
| N08 | Neutral | 3.06 (0.65) | 2.84 (0.74) | Anna | CC BY 2.0^c^ | https://goo.gl/9DcLPX | - |
| N09 | Neutral | 3.18 (0.44) | 2.92 (0.49) | Antonio Campos Ruiz | CC BY 2.0^c^ | https://goo.gl/LBNTM1 | Yes |
| N10 | Neutral | 3.42 (0.67) | 2.50 (0.74) | Sunchild57 Photography | CC BY-NC-SA 2.0^e^ | https://goo.gl/pS18sf | - |
| N11 | Neutral | 3.04 (0.28) | 2.92 (0.44) | Ateliê Maracanã | CC BY-NC 2.0^a^ | https://goo.gl/fWsRar | - |
| N12 | Neutral | 3.66 (0.75) | 2.42 (0.84) | Sylvio Bazote | CC BY-NC-SA 2.0^e^ | https://goo.gl/CHPjyd | Yes |
| N13 | Neutral | 3.22 (0.51) | 2.72 (0.67) | Tania Piza | CC BY 2.0^c^ | https://goo.gl/fegQL5 | - |
| N14 | Neutral | 3.04 (0.20) | 2.94 (0.42) | Thomas Quine | CC BY 2.0^c^ | https://goo.gl/UqsgMX | - |
| N15 | Neutral | 3.30 (0.58) | 2.74 (0.57) | Bob Jagendorf | CC BY-NC 2.0^a^ | https://goo.gl/iScWDS | - |
| N16 | Neutral | 3.08 (0.34) | 2.82 (0.60) | Bruna Alves | CC BY-NC-SA 2.0^e^ | https://goo.gl/AgYWYu | Yes |
| N17 | Neutral | 3.00 (0.20) | 2.94 (0.37) | El Patojo | CC BY-NC-SA 2.0^e^ | https://goo.gl/s2kzLq | - |
| N18 | Neutral | 3.08 (0.34) | 2.82 (0.48) | Vincent Ma | CC BY-NC-SA 2.0^e^ | https://goo.gl/hYtyt2 | - |
| N19 | Neutral | 3.26 (0.53) | 2.58 (0.70) | Vinicius Pinheiro | CC BY-SA 2.0^b^ | https://goo.gl/KFiWPr | - |
| N20 | Neutral | 3.12 (0.39) | 2.74 (0.63) | El Patojo | CC BY-NC-SA 2.0^e^ | https://goo.gl/RJQMTq | - |
| N21 | Neutral | 3.12 (0.39) | 2.80 (0.54) | swirlingthoughts | CC BY-NC 2.0^a^ | https://goo.gl/YPGPwV | - |
| N22 | Neutral | 3.26 (0.60) | 2.82 (0.63) | Clément Alloing | CC BY-NC-ND 2.0^d^ | https://goo.gl/L7f9Yr | - |
| N23 | Neutral | 3.24 (0.52) | 2.62 (0.70) | skepticalview | CC BY-NC-ND 2.0^d^ | https://goo.gl/MM74Zm | - |
| N24 | Neutral | 3.04 (0.20) | 2.92 (0.44) | danSelva | CC BY 2.0^c^ | https://goo.gl/Ar92Pm | - |
| N25 | Neutral | 3.14 (0.41) | 2.82 (0.52) | Douglas Porter | CC BY-NC-ND 2.0^d^ | https://goo.gl/8e2Nis | - |
| **Picture identification** | **Category** | **Descriptive statistics** | | **Credits** | **License** | **URL** | **Modifications** |
|  |  | **Valence** | **Arousal** |  |  |  |  |
|  |  | ***M* (*SD*)** | ***M* (*SD*)** |  |  |  |  |
| N26 | Neutral | 3.12 (0.39) | 2.88 (0.48) | El Patojo | CC BY-NC-SA 2.0^e^ | https://goo.gl/tc5nNq | - |
| N27 | Neutral | 3.16 (0.42) | 2.68 (0.71) | El Patojo | CC BY-NC-SA 2.0^e^ | https://goo.gl/JV9F7o | - |
| N28 | Neutral | 3.06 (0.37) | 2.88 (0.63) | nebojsa mladjenovic | CC BY-NC-ND 2.0^d^ | https://goo.gl/r6UFr8 | - |
| N29 | Neutral | 3.02 (0.14) | 2.92 (0.34) | Fábio Biff | CC BY-NC 2.0^a^ | https://goo.gl/kbEfRQ | Yes |
| N30 | Neutral | 3.02 (0.32) | 2.86 (0.61) | Fábio Biff | CC BY-NC 2.0^a^ | https://goo.gl/Ms3df7 | Yes |
| N31 | Neutral | 3.10 (0.36) | 2.82 (0.52) | Fellipe Cicconi | CC BY-NC 2.0^a^ | https://goo.gl/ZeHfeC | Yes |
| N32 | Neutral | 3.08 (0.27) | 2.76 (0.63) | Norman Hooper | CC BY-SA 2.0^b^ | https://goo.gl/Y9R7Dy | - |
| N33 | Neutral | 3.16 (0.42) | 2.80 (0.67) | Francisco Antunes | CC BY 2.0^c^ | https://goo.gl/BMkcwL | - |
| N34 | Neutral | 3.36 (0.63) | 2.52 (0.79) | John Keogh | CC BY-NC 2.0^a^ | https://goo.gl/oagRgV | - |
| N35 | Neutral | 3.20 (0.57) | 2.64 (0.78) | grytr | CC BY-NC-ND 2.0^d^ | https://goo.gl/Qo5k1N | - |
| N36 | Neutral | 3.34 (0.59) | 2.48 (0.79) | Karolina Lubryczynska | CC BY-NC-ND 2.0^d^ | https://goo.gl/xKtkuj | - |
| N37 | Neutral | 3.04 (0.35) | 2.90 (0.42) | Klaus Post | CC BY 2.0^c^ | https://goo.gl/auy3ec | - |
| N38 | Neutral | 3.34 (0.59) | 2.50 (0.79) | Klaus Post | CC BY 2.0^c^ | https://goo.gl/G79P2k | - |
| N39 | Neutral | 3.10 (0.42) | 2.76 (0.72) | Tatiana Roza | CC BY-NC-ND 2.0^d^ | https://goo.gl/5fKXtN | - |
| N40 | Neutral | 3.62 (0.67) | 2.16 (0.87) | Manuel W | CC BY-NC 2.0^a^ | https://goo.gl/3H8HiW | - |
| N41 | Neutral | 3.30 (0.61) | 2.60 (0.83) | Louis Kreusel | CC BY-NC 2.0^a^ | https://goo.gl/T62Pvw | - |
| N42 | Neutral | 3.22 (0.58) | 2.72 (0.61) | Marcelo Paes | CC BY-NC 2.0^a^ | https://goo.gl/M9e7E7 | - |
| N43 | Neutral | 3.16 (0.47) | 2.78 (0.55) | Mariko Watanabe | CC BY-NC-ND 2.0^d^ | https://goo.gl/RkrCMR | - |
| N44 | Neutral | 3.04 (0.28) | 2.98 (0.38) | Wade Morgen | CC BY-NC 2.0^a^ | https://goo.gl/oxKU2m | - |
| N45 | Neutral | 2.98 (0.38) | 2.96 (0.57) | Karen Bryan | CC BY-ND 2.0^f^ | https://goo.gl/yN3iBs | - |
| N46 | Neutral | 3.00 (0.20) | 2.92 (0.44) | Luiz Claudio | CC BY-NC 2.0^a^ | https://goo.gl/v99HyH | Yes |
| N47 | Neutral | 2.96 (0.28) | 3.06 (0.62) | go elsewhere... | CC BY-NC 2.0^a^ | https://goo.gl/8NdgVF | - |
| N48 | Neutral | 3.00 (0.29) | 2.96 (0.61) | MySafetySign.com | CC BY 2.0^c^ | https://goo.gl/qtqoMA | - |
| N49 | Neutral | 2.98 (0.25) | 2.96 (0.28) | Beth Bryda | CC BY 2.0^c^ | https://goo.gl/MsYwKS | - |
| N50 | Neutral | 3.02 (0.14) | 2.96 (0.35) | Turnstone Furniture | CC BY-ND 2.0^f^ | https://goo.gl/LBofoK | - |
| FL01 | Flood | 1.44 (0.54) | 4.28 (0.83) | Nathalie Gutierrez/Milton Jung | CC BY 2.0^c^ | https://goo.gl/chpYFH | - |
| FL02 | Flood | 1.94 (0.94) | 4.18 (0.69) | Trokilinochchi | CC BY 2.0^c^ | https://goo.gl/9a3RiQ | - |
| FL03 | Flood | 1.70 (0.54) | 4.18 (0.80) | shelly-jo | CC BY-NC-ND 2.0^d^ | https://goo.gl/eau7C2 | - |
| **Picture identification** | **Category** | **Descriptive statistics** | | **Credits** | **License** | **URL** | **Modifications** |
|  |  | **Valence** | **Arousal** |  |  |  |  |
|  |  | ***M* (*SD*)** | ***M* (*SD*)** |  |  |  |  |
| FL04 | Flood | 2.08 (0.49) | 3.80 (0.76) | Mark Robinson | CC BY-NC 2.0^a^ | https://goo.gl/bzJcQ4 | - |
| FL05 | Flood | 1.94 (0.94) | 4.06 (0.96) | K38 Rescue | CC BY-NC-ND 2.0^d^ | https://goo.gl/9vYWhd | - |
| FL06 | Flood | 1.20 (0.45) | 4.66 (0.66) | Kelly Garbato | CC BY-NC 2.0^a^ | https://goo.gl/ALvca7 | - |
| FL07 | Flood | 2.12 (1.24) | 4.30 (0.79) | PINKÉ | CC BY-NC 2.0^a^ | https://goo.gl/uLXzao | - |
| FL08 | Flood | 1.68 (0.59) | 4.30 (0.79) | Trokilinochchi | CC BY 2.0^c^ | https://goo.gl/812pDC | - |
| FL09 | Flood | 1.94 (0.47) | 3.90 (0.71) | johndal | CC BY 2.0^c^ | https://goo.gl/4VAwuB | - |
| FL10 | Flood | 1.96 (0.90) | 4.24 (0.85) | Oxfam International | CC BY-NC-ND 2.0^d^ | https://goo.gl/Aw5dso | - |
| FL11 | Flood | 2.48 (1.11) | 3.86 (0.86) | Manchester Fire | CC BY-NC 2.0^a^ | https://goo.gl/JgcycU | - |
| FL12 | Flood | 1.58 (0.58) | 4.26 (0.69) | DVIDSHUB | CC BY 2.0^c^ | https://goo.gl/2DA7d8 | - |
| FL13 | Flood | 2.00 (0.45) | 3.80 (0.64) | poulsbo | CC BY-NC-ND 2.0^d^ | https://goo.gl/8Akk15 | - |
| FL14 | Flood | 1.90 (0.54) | 4.06 (0.74) | Ronaldo Alexandre/Cassimano | CC BY-NC-SA 2.0^e^ | https://goo.gl/oaEVQc | - |
| FL15 | Flood | 1.60 (0.73) | 4.36 (0.78) | Trokilinochchi | CC BY 2.0^c^ | https://goo.gl/pPmBsL | - |
| FL16 | Flood | 2.14 (0.50) | 3.66 (0.63) | Peter Kelly | CC BY-NC 2.0^a^ | https://goo.gl/98JbNP | - |
| FL17 | Flood | 2.18 (1.02) | 4.10 (0.68) | The National Guard | CC BY 2.0^c^ | https://goo.gl/4sPMq4 | - |
| FL18 | Flood | 1.88 (0.44) | 3.98 (0.62) | Wiesbaden112.de | CC BY-NC-ND 2.0^d^ | https://goo.gl/JNpHBF | - |
| FL19 | Flood | 1.70 (0.97) | 4.42 (0.73) | United Nations Photo | CC BY-NC-ND 2.0^d^ | https://goo.gl/JtwJ5u | - |
| FL20 | Flood | 1.46 (0.54) | 4.56 (0.54) | AmazonCARES | CC BY 2.0^c^ | https://goo.gl/oWfvGb | - |
| FL21 | Flood | 1.62 (0.60) | 4.38 (0.70) | Trokilinochchi | CC BY 2.0^c^ | https://goo.gl/6sQec9 | - |
| FL22 | Flood | 1.68 (0.55) | 4.24 (0.66) | Ronaldo Alexandre/Cassimano | CC BY-NC-SA 2.0^e^ | https://goo.gl/tp9DtS | - |
| FL23 | Flood | 1.70 (0.58) | 4.30 (0.65) | Trokilinochchi | CC BY 2.0^c^ | https://goo.gl/oNdVWd | - |
| FL24 | Flood | 1.84 (0.62) | 4.14 (0.76) | Fernando Stankuns | CC BY-NC-SA 2.0^e^ | https://goo.gl/GNYvt5 | - |
| FL25 | Flood | 1.82 (0.52) | 4.16 (0.71) | jonathanvlarocca | CC BY 2.0^c^ | https://goo.gl/D62gCd | - |
| FL26 | Flood | 1.78 (0.55) | 4.26 (0.69) | Charles Wiriawan | CC BY-NC-ND 2.0^d^ | https://goo.gl/U4wvZn | - |
| FL27 | Flood | 1.68 (0.55) | 4.16 (0.74) | Trokilinochchi | CC BY 2.0^c^ | https://goo.gl/dPr9ds | - |
| FL28 | Flood | 1.34 (0.56) | 4.58 (0.67) | Mathias Fingermann | CC BY-NC 2.0^a^ | https://goo.gl/4oub8j | - |
| FL29 | Flood | 2.00 (1.18) | 4.32 (0.87) | United Nations Photo | CC BY-NC-ND 2.0^d^ | https://goo.gl/9tAFLw | - |
| FL30 | Flood | 2.00 (0.40) | 3.94 (0.65) | johndal | CC BY 2.0^c^ | https://goo.gl/aAeUL5 | - |
| FL31 | Flood | 1.68 (0.59) | 4.20 (0.70) | Milton Jung | CC BY 2.0^c^ | https://goo.gl/SCLZru | - |
| **Picture identification** | **Category** | **Descriptive statistics** | | **Credits** | **License** | **URL** | **Modifications** |
|  |  | **Valence** | **Arousal** |  |  |  |  |
|  |  | ***M* (*SD*)** | ***M* (*SD*)** |  |  |  |  |
| FL32 | Flood | 1.80 (0.50) | 4.02 (0.69) | Milton Jung | CC BY-NC 2.0^a^ | https://goo.gl/bVgfxm | - |
| FL33 | Flood | 2.12 (1.04) | 4.18 (0.85) | Trokilinochchi | CC BY 2.0^c^ | https://goo.gl/AyRRXH | - |
| FL34 | Flood | 1.68 (0.51) | 4.26 (0.75) | Trokilinochchi | CC BY 2.0^c^ | https://goo.gl/EMDa19 | - |
| FL35 | Flood | 1.78 (0.51) | 4.16 (0.77) | Cassimano | CC BY-NC-SA 2.0^e^ | https://goo.gl/zBn2tf | - |
| FL36 | Flood | 1.84 (0.55) | 4.08 (0.78) | Fernando Stankuns | CC BY-NC-SA 2.0^e^ | https://goo.gl/LSWHin | Yes |
| FL37 | Flood | 1.62 (0.53) | 4.44 (0.68) | Milton Jung / Cátia Toffoletto | CC BY 2.0^c^ | https://goo.gl/FzMTL6 | - |
| FL38 | Flood | 1.86 (0.61) | 4.18 (0.63) | Milton Jung | CC BY-NC 2.0^a^ | https://goo.gl/Fu4Mqr | - |
| FL39 | Flood | 1.96 (0.45) | 3.84 (0.74) | shelly-jo | CC BY-NC-ND 2.0^d^ | https://goo.gl/Y1TDDu | - |
| FL40 | Flood | 1.96 (0.49) | 3.86 (0.76) | johndal | CC BY 2.0^c^ | https://goo.gl/1v73D5 | - |
| FL41 | Flood | 1.96 (0.61) | 3.78 (0.79) | Tejvan Pettinger | CC BY 2.0^c^ | https://goo.gl/bGLwmo | - |
| FL42 | Flood | 1.30 (0.46) | 4.70 (0.54) | Cátia Toffoletto/Milton Jung | CC BY 2.0^c^ | https://goo.gl/1mUHEa | - |
| FL43 | Flood | 1.66 (0.77) | 4.28 (0.76) | Trokilinochchi | CC BY 2.0^c^ | https://goo.gl/W7posr | - |
| FL44 | Flood | 1.98 (0.65) | 3.86 (0.70) | Liz Smith | CC BY-NC-ND 2.0^d^ | https://goo.gl/mubiR9 | - |
| FL45 | Flood | 1.74 (0.60) | 4.10 (0.79) | EU Civil Protection and Humanitarian Aid Operations | CC BY-SA 2.0^b^ | https://goo.gl/FmaJxg | - |
| FL46 | Flood | 1.92 (0.53) | 3.96 (0.73) | johndal | CC BY 2.0^c^ | https://goo.gl/L1YKgA | - |
| FL47 | Flood | 1.72 (0.50) | 4.34 (0.75) | Big Swede Guy | CC BY-NC-ND 2.0^d^ | https://goo.gl/25NR2f | - |
| FL48 | Flood | 1.58 (0.67) | 4.42 (0.67) | Trokilinochchi | CC BY 2.0^c^ | https://goo.gl/FUABBo | - |
| FL49 | Flood | 1.88 (0.59) | 4.18 (0.45) | waldopepper | CC BY-NC 2.0^a^ | https://goo.gl/LbKQ6o | - |
| FL50 | Flood | 2.00 (0.45) | 3.82 (0.66) | johndal | CC BY 2.0^c^ | https://goo.gl/bdYjSY | - |
| DR01 | Drought | 2.10 (0.65) | 3.82 (0.75) | Padre Djacy Brasileiro | CC BY-NC-ND 2.0^d^ | https://goo.gl/mDELhU | - |
| DR02 | Drought | 1.88 (0.77) | 3.86 (0.95) | Asian Development Bank | CC BY-NC-ND 2.0^d^ | https://goo.gl/4YaUMN | - |
| DR03 | Drought | 1.82 (0.66) | 3.98 (0.80) | EU Civil Protection and Humanitarian Aid Operations | CC BY-NC-ND 2.0^d^ | https://goo.gl/GBme8F | - |
| DR04 | Drought | 2.32 (0.87) | 3.50 (0.84) | Chiranjit Ojha | CC BY-NC-SA 2.0^e^ | https://goo.gl/pEyFKm | - |
| DR05 | Drought | 2.10 (0.71) | 3.60 (0.83) | David Kelleher | CC BY-NC-ND 2.0^d^ | https://goo.gl/LPSkFk | - |
| DR06 | Drought | 1.70 (1.00) | 4.42 (0.80) | Andrew Heavens | CC BY-NC-ND 2.0^d^ | https://goo.gl/8tACyF | - |
| DR07 | Drought | 1.90 (0.58) | 3.86 (0.78) | David Kelleher | CC BY-NC-ND 2.0^d^ | https://goo.gl/mKQTe6 | - |
| **Picture identification** | **Category** | **Descriptive statistics** | | **Credits** | **License** | **URL** | **Modifications** |
|  |  | **Valence** | **Arousal** |  |  |  |  |
|  |  | ***M* (*SD*)** | ***M* (*SD*)** |  |  |  |  |
| DR08 | Drought | 1.06 (0.24) | 4.86 (0.35) | AlunMcDonald/Oxfam | CC BY-NC-ND 2.0^d^ | https://goo.gl/UVR57E | - |
| DR09 | Drought | 1.96 (0.73) | 3.98 (0.96) | Anna Ridout/Oxfam | CC BY-NC-ND 2.0^d^ | https://goo.gl/mysDjp | - |
| DR10 | Drought | 1.68 (0.51) | 4.20 (0.73) | David Kelleher | CC BY-NC-ND 2.0^d^ | https://goo.gl/vp6Hs6 | - |
| DR11 | Drought | 1.14 (0.35) | 4.82 (0.44) | EU Civil Protection and Humanitarian Aid Operations | CC BY-NC-ND 2.0^d^ | https://goo.gl/nM7Z3D | - |
| DR12 | Drought | 2.50 (0.65) | 3.28 (0.70) | EC/ECHO Maria Olsen | CC BY-NC-ND 2.0^d^ | https://goo.gl/JxRtuH | - |
| DR13 | Drought | 2.58 (0.93) | 3.38 (0.88) | Jane | CC BY-NC-ND 2.0^d^ | https://goo.gl/SHWpFs | - |
| DR14 | Drought | 1.74 (0.72) | 4.44 (0.61) | Padre Djacy Brasileiro | CC BY-NC-ND 2.0^d^ | https://goo.gl/FwcswZ | - |
| DR15 | Drought | 1.16 (0.37) | 4.84 (0.42) | Padre Djacy Brasileiro | CC BY-NC-ND 2.0^d^ | https://goo.gl/Knwzbs | - |
| DR16 | Drought | 2.38 (0.67) | 3.62 (0.67) | Julio Cezar Winkler | CC BY-NC-SA 2.0^e^ | https://goo.gl/oHv2qX | - |
| DR17 | Drought | 1.82 (0.63) | 3.94 (0.82) | Padre Djacy Brasileiro | CC BY-NC-ND 2.0^d^ | https://goo.gl/E9jPPT | - |
| DR18 | Drought | 1.68 (0.77) | 4.32 (0.62) | Padre Djacy Brasileiro | CC BY-NC-ND 2.0^d^ | https://goo.gl/dvtLXU | - |
| DR19 | Drought | 1.94 (0.65) | 3.86 (0.86) | Marufish | CC BY-SA 2.0^b^ | https://goo.gl/fna1ws | - |
| DR20 | Drought | 1.32 (0.47) | 4.60 (0.54) | Luca De Vito | CC BY-NC-ND 2.0^d^ | https://goo.gl/Amev23 | - |
| DR21 | Drought | 1.92 (0.60) | 3.88 (0.87) | Modern Event Preparedness | CC BY 2.0^c^ | https://goo.gl/yE4bBp | - |
| DR22 | Drought | 1.96 (0.61) | 3.94 (0.77) | Padre Djacy Brasileiro | CC BY-NC-ND 2.0^d^ | https://goo.gl/Q8D22z | - |
| DR23 | Drought | 1.98 (0.77) | 3.98 (0.85) | Pablo Tosco/Oxfam | CC BY-NC-ND 2.0^d^ | https://goo.gl/PwNUdt | - |
| DR24 | Drought | 2.40 (0.73) | 3.46 (0.84) | Otávio Nogueira | CC BY 2.0^c^ | https://goo.gl/ubbLLb | - |
| DR25 | Drought | 1.08 (0.27) | 4.86 (0.35) | Padre Djacy Brasileiro | CC BY-NC-ND 2.0^d^ | https://goo.gl/W3315G | - |
| DR26 | Drought | 2.08 (0.53) | 3.72 (0.76) | Padre Djacy Brasileiro | CC BY-NC-ND 2.0^d^ | https://goo.gl/jJmw2v | - |
| DR27 | Drought | 1.72 (0.57) | 4.30 (0.81) | Pablo Tosco/Oxfam | CC BY-NC-ND 2.0^d^ | https://goo.gl/S2K3M1 | - |
| DR28 | Drought | 1.62 (0.64) | 4.32 (0.71) | Padre Djacy Brasileiro | CC BY-NC-ND 2.0^d^ | https://goo.gl/obgN4w | - |
| DR29 | Drought | 2.28 (0.73) | 3.58 (0.95) | Otávio Nogueira | CC BY 2.0^c^ | https://goo.gl/GAChPR | - |
| DR30 | Drought | 2.38 (0.78) | 3.46 (0.91) | Padre Djacy Brasileiro | CC BY-NC-ND 2.0^d^ | https://goo.gl/F15i2f | - |
| DR31 | Drought | 2.66 (0.75) | 3.16 (0.91) | Padre Djacy Brasileiro | CC BY-NC-ND 2.0^d^ | https://goo.gl/qZdcHP | - |
| DR32 | Drought | 1.08 (0.27) | 4.88 (0.33) | Padre Djacy Brasileiro | CC BY-NC-ND 2.0^d^ | https://goo.gl/SAEcyV | - |
| DR33 | Drought | 1.92 (0.63) | 3.84 (0.91) | Padre Djacy Brasileiro | CC BY-NC-ND 2.0^d^ | https://goo.gl/1pV5o8 | - |
| DR34 | Drought | 2.36 (0.69) | 3.50 (0.95) | Otávio Nogueira | CC BY 2.0^c^ | https://goo.gl/n7DVht | - |
| **Picture identification** | **Category** | **Descriptive statistics** | | **Credits** | **License** | **URL** | **Modifications** |
|  |  | **Valence** | **Arousal** |  |  |  |  |
|  |  | ***M* (*SD*)** | ***M* (*SD*)** |  |  |  |  |
| DR35 | Drought | 2.08 (0.63) | 3.78 (0.82) | Padre Djacy Brasileiro | CC BY-NC-ND 2.0^d^ | https://goo.gl/ps3TyG | - |
| DR36 | Drought | 1.84 (0.71) | 4.06 (0.94) | Padre Djacy Brasileiro | CC BY-NC-ND 2.0^d^ | https://goo.gl/WLHMV4 | - |
| DR37 | Drought | 2.20 (0.67) | 3.68 (0.87) | Otávio Nogueira | CC BY 2.0^c^ | https://goo.gl/g8vFyX | - |
| DR38 | Drought | 2.28 (0.70) | 3.64 (0.88) | Tim J Keegan | CC BY-SA 2.0^b^ | https://goo.gl/YMWWYL | - |
| DR39 | Drought | 1.66 (0.69) | 4.54 (0.68) | UNICEF Ethiopia | CC BY-NC-ND 2.0^d^ | https://goo.gl/ntnHW1 | - |
| DR40 | Drought | 2.12 (0.80) | 3.60 (0.95) | BRJ INC. | CC BY-NC-ND 2.0^d^ | https://goo.gl/N4MVrc | - |
| DR41 | Drought | 2.14 (0.78) | 3.74 (0.88) | Otávio Nogueira | CC BY 2.0^c^ | https://goo.gl/KVY4QS | - |
| DR42 | Drought | 1.10 (0.30) | 4.86 (0.35) | Oxfam International | CC BY-NC-ND 2.0^d^ | https://goo.gl/ySST4A | - |
| DR43 | Drought | 1.48 (0.65) | 4.44 (0.79) | DFID - UK Department for International Development | CC BY 2.0^c^ | https://goo.gl/XX5eJG | - |
| DR44 | Drought | 1.58 (0.70) | 4.44 (0.76) | Riccardo Romano | CC BY-NC-ND 2.0^d^ | https://goo.gl/fy1ynx | - |
| DR45 | Drought | 1.28 (0.54) | 4.62 (0.75) | Oxfam International | CC BY-NC-ND 2.0^d^ | https://goo.gl/C89qRu | - |
| DR46 | Drought | 2.10 (0.76) | 3.80 (0.90) | Anita Ritenour | CC BY 2.0^c^ | https://goo.gl/CJGR7Q | - |
| DR47 | Drought | 1.06 (0.24) | 4.74 (0.69) | UNICEF Ethiopia | CC BY-NC-ND 2.0^d^ | https://goo.gl/nK9yKa | - |
| DR48 | Drought | 1.74 (0.69) | 4.26 (0.72) | UNICEF Ethiopia | CC BY-NC-ND 2.0^d^ | https://goo.gl/axSaHV | - |
| DR49 | Drought | 2.66 (0.63) | 3.10 (0.76) | Kevin Dooley | CC BY 2.0^c^ | https://goo.gl/EhBPRj | - |
| DR50 | Drought | 1.74 (0.60) | 4.20 (0.86) | Padre Djacy Brasileiro | CC BY-NC-ND 2.0^d^ | https://goo.gl/6zzr2G | - |

^a^Attribution-NonCommercial 2.0 Generic (https://goo.gl/u216xp)

^b^Attribution-ShareAlike 2.0 Generic (https://goo.gl/xSmtBZ)

^c^Attribution 2.0 Generic (https://goo.gl/wmY3Rk)

^d^Attribution-NonCommercial-NoDerivs 2.0 Generic (https://goo.gl/imfcio)

^e^Attribution-NonCommercial-ShareAlike 2.0 Generic (https://goo.gl/zr9Rbn)

## ^f^Attribution-NoDerivs 2.0 Generic (https://goo.gl/mwwyUJ)
